# Evolution of Allorecognition in the Tunicata

**DOI:** 10.3390/biology9060129

**Published:** 2020-06-16

**Authors:** Marie L. Nydam

**Affiliations:** Math and Science Program, Soka University of America, 1 University Drive, Aliso Viejo, CA 92656, USA; mnydam@soka.edu

**Keywords:** colonial invertebrate, self-incompatibility, fusion, tunicate, ascidian, Stolidobranchia, Phlebobranchia, Aplousobranchia

## Abstract

Allorecognition, the ability to distinguish self or kin from unrelated conspecifics, plays several important biological roles in invertebrate animals. Two of these roles include negotiating limited benthic space for colonial invertebrates, and inbreeding avoidance through self-incompatibility systems. Subphylum Tunicata (Phylum Chordata), the sister group to the vertebrates, is a promising group in which to study allorecognition. Coloniality has evolved many times independently in the tunicates, and the best known invertebrate self-incompatibility systems are in tunicates. Recent phylogenomic studies have coalesced around a phylogeny of the Tunicata as well as the Order Stolidobranchia within the Tunicata, providing a path forward for the study of allorecognition in this group.

## 1. Allorecognition in Invertebrate Animals

Allorecognition, broadly defined as the ability to recognize self from non-self, is widespread across the tree of life [1]. Allorecognition may have initially evolved as a way to alert the immune system to a pathogen or parasite, as innate immunity is evolutionarily conserved [2,3]. Interspecific immune-related systems seem to be the foundation upon which intraspecific recognition systems evolved [4]. Intraspecific recognition systems tied to disease resistance include MHC-mediated mate choice in many vertebrates (reviewed in [5]), gametic interactions in plants [6,7] and invertebrates [8,9], and fusion in invertebrates [10,11].

This review focuses on intraspecific recognition systems and will therefore use a narrower definition of allorecognition: the ability to distinguish self or kin from unrelated conspecifics. In invertebrate animals, allorecognition plays two important biological roles: negotiating space and regulating mating.

In space-limited benthic habitats, colonial growth forms are common, and colonies often encounter conspecifics [12,13]. There are at least two explanations for why allorecognition systems evolved in the context of coloniality: (1) to re-connect sections of colonies that were separated by predation, disease, or storms [14] (2) as an adaptation to stem cell parasitism [1]. Stem cell parasitism is a potential outcome of fusion between two colonies, where the stem cells of a winning colony invade the losing colony [15]. Stem cell parasitism often results in a precipitous decline in fitness for the losing colony, as usually only the winner’s genotype is represented in the offspring of the fused colony [15]. If fusions are restricted to kin, then the fitness declines experienced by losing colonies are mitigated [1].

Allorecognition is also linked to self-incompatibility mating systems in marine invertebrates. Self-incompatibility systems prevent inbreeding depression, and may be selected for in marine invertebrates that broadcast spawn (release sperm and eggs, fertilization occurs in the water) or spermcast (release sperm only, fertilization occurs inside the maternal animal) [16]. In these species, there is no copulatory behavior through which selection can favor individuals who choose unrelated mates [17].

Inbreeding prevalence in marine invertebrates is more common than previously thought [17]. The percentage of F_IS_ values greater than 0.1 in broadcast or spermcast spawning species is 62% (raw data obtained from [17]). In these species, lowering the fitness cost of inbreeding through the purging of recessive deleterious alleles [18] may have evolved as an alternative to self-incompatibility systems. However, 38% of broadcasting and spermcasting species have F_IS_ values less than 0.1 (raw data obtained from [17]). In these outcrossing species, selection for inbreeding avoidance, including self-incompatibility systems, should be strong.

In this review, allorecognition will be explored in the Tunicata. Subphylum Tunicata (Phylum Chordata), the sister group to the vertebrates, is a promising group in which to study allorecognition. One of the best-known systems of allorecognition, and furthermore one of the only invertebrate allorecognition systems for which the genetic underpinnings are known, occurs in the botryllid ascidians (family Styelidae). The evolution of allorecognition can be examined in the context of coloniality and mating systems. The tunicates contain both colonial and solitary species, and coloniality has evolved many times independently [19]. The best known invertebrate self-incompatibility systems are in tunicates: in the phlebobranch ascidian *Ciona robusta* [20] and the stolidobranch ascidians *Halocynthia aurantium* and *Halocynthia roretzi* [21,22].

## 2. Relationships within the Subphylum Tunicata

Tunicate biologists have long debated the evolution of coloniality, and therefore allorecognition, owing to uncertainties in the phylogenetic relationships within this group. Recent phylogenomic studies have coalesced around a phylogeny of the Tunicata, providing a path forward for the study of allorecognition in this group.

**Figure 1:**
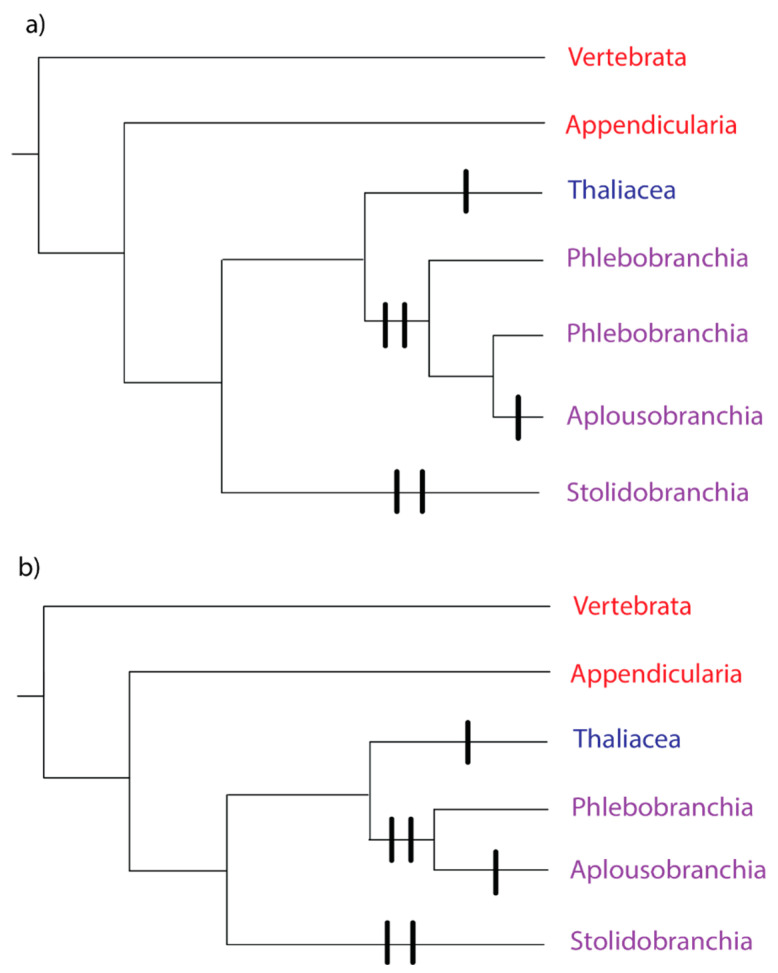
Phylogeny of the Subphylum Tunicata, based on [31,32]. The tree includes Subphylum Vertebrata as the outgroup. Red text denotes a group where all species have solitary growth forms, blue text denotes a group where all species have colonial growth forms, and purple text denotes a group with both solitary and colonial growth forms. Vertical bars represent independent evolutions of the colonial growth form. (**a**) Phlebobranchia paraphyletic, (**b**) Phlebobranchia monophyletic.

Subphylum Tunicata comprises Class Appendicularia (pelagic larvaceans), Class Ascidiacea (benthic ascidians), and Class Thaliacea (pelagic doliolids, pyrosomes, and salps) [23]. For the last 20 years, phylogenies based on 18S rRNA [24,25,26,27] and mitochondrial genomes [28,29,30] have consistently agreed that Class Ascidiacea is paraphyletic with respect to Class Thaliacea: thaliaceans belong in a monophyletic group with the ascidian orders Phlebobranchia and Aplousobranchia (Figure 1). This thaliacean/phlebobranch/aplousobranch clade is sister to a clade comprising the ascidian order Stolidobranchia (Figure 1). But the relationships within the thaliacean/phlebobranch/aplousobranch clade, and the position of Class Appendicularia, were unresolved.

Recent nuclear phylogenomic studies [31,32] have made progress on the unresolved nodes. In both studies, the thaliaceans are the outgroup to the phlebobranch/aplousobranch clade, with Appendicularia as the outgroup to the rest of the Tunicata (Figure 1). The remaining unanswered question is whether Phlebobranchia is paraphyletic with respect to Aplousobranchia (Figure 1a), or whether Phlebobranchia and Aplousobranchia are monophyletic groups (Figure 1b). The majority of the analyses in [32] recovered phlebobranch paraphyly, but with variable support. A subset of the 50 genes with the most stable amino acid frequencies across the tree produced a monophyletic and well-supported Phlebobranchia [32]. The analyses in [31] resulted in a paraphyletic Phlebobranchia, but with no statistical support.

## 3. Evolution of Allorecognition within the Subphylum Tunicata: Negotiating Space

As discussed above, allorecognition in many colonial invertebrates likely evolved as a response to competitive interactions in crowded benthic environments [13]. Therefore, the evolution of allorecognition systems governing fusion is closely linked to the evolution of coloniality. Within the Subphylum Tunicata, only Class Ascidiacea has adopted a benthic lifestyle, so ascidians will be the focus of this section. For each ascidian order (Aplousobranchia, Phlebobranchia, Stolidobranchia), the evolution of coloniality will be reviewed, followed by the evolution of allorecognition.

Tunicata and Vertebrata together form the clade Olfactores [33], and the ancestor of this clade most likely had a solitary growth form and a benthic lifestyle [34]. The tunicate ancestor was also likely to be solitary (Figure 1). Coloniality likely evolved independently on the lineage, leading to Class Thaliacea (Figure 1), because the asexual buds develop from an endostyle outgrowth, a mechanism distinct from other tunicates [35].

### 3.1. Order Stolidobranchia

#### 3.1.1. Coloniality in Order Stoliobranchia

**Figure 2:**
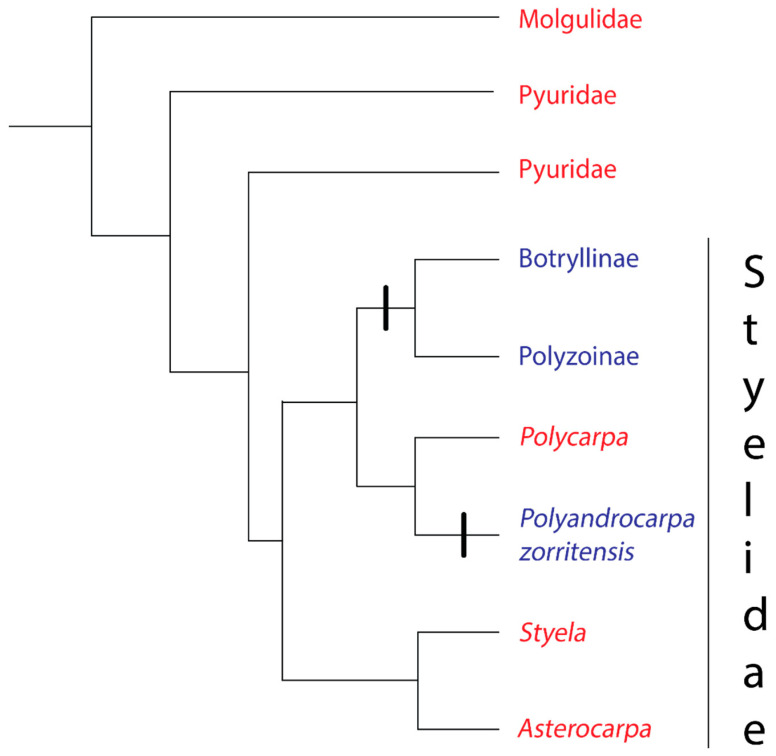
Phylogeny of the Order Stolidobranchia, based on [36]. The order contains three families: Molgulidae, Pyuridae, and Styelidae. Red text denotes a group where all species have solitary growth forms, Blue text denotes a group where all species have colonial growth forms.

The families at the base of the stolidobranch tree (Molgulidae and Pyuridae) are solitary (Figure 2). The third family in the Order Stolidobranchia, Styelidae, contains both solitary and colonial species [23]. Within the Styelidae, coloniality evolved twice, once in the ancestor of the Botryllinae + Polyzoinae clade [25,36] and again in *Polyandrocarpa zorritensis* [36] (Figure 2). Convergent rather than homologous evolution of coloniality in the family Styelidae is indicated by two strong lines of evidence. First, Botryllinae + Polyzoinae create colonies through peribranchial budding [36,37], with Botryllinae + *Symplegma* in the Polyzoinae also capable of vascular budding [38,39], whereas *P.zorritensis* is unique among the tunicates in its use of vasal budding [40]. Second, an evolution of coloniality in the most recent common ancestor of Botryllinae, Polyzoinae, and *P.zorritensis* would contradict the principle of parsimony, requiring four to six character state changes [36].

#### 3.1.2. Allorecognition in Order Stolidobranchia

While there is evidence for tunic fusion in two species of solitary stolidobranchs (*Molgula complanata* and *Styela plicata*) it is unclear whether the fusion involves the creation of a common vascular system and whether the fusion is discriminatory [41,42]. Setting aside these uncertain cases, allorecognition evolved at least twice: at least once the base of the Polyzoinae/Botryllinae clade when coloniality evolved in this group, and again when coloniality evolved in *P. zorritensis* (Figure 2).

Allorecognition occurs in Symplegma reptans in the subfamily Polyzoinae [43] and in 10 species in the subfamily Botryllinae: Botrylloides diegensis, Botrylloides fuscus, Botrylloides lentus, Botrylloides simodensis, Botrylloides violaceus, Botryllus delicatus, Botryllus primigenus, Botryllus promiscuus, Botryllus puniceus, and Botryllus scalaris [44,45,46,47,48,49,50,51,52]. Symplegma and botryllids have similarities in their rejection reactions [43]. It would be interesting to determine if the genes involved in allorecognition in botryllids [53] are found in S. reptans. This information could help determine whether allorecognition evolved at the base of the Polyzoinae/Botryllinae clade (as is posited for coloniality in [36]), or separately in each subfamily.

The evolution of allorecognition within the subfamily Botryllinae is well understood, owing to 60 years of study by Oka, Saito, Watanabe and students. Each botryllid species studied thus far exhibits one of three types of allogeneic rejection responses, which vary in the amount of physical interaction between colonies before points of rejection are formed [10]. In Type I, the outer coverings (tunics) fuse in limited areas, whereas the complete fusion of the outer tunics occurs in Type II and III [10]. In Type III but not Type I or II responses, the vascular systems fuse prior to rejection [10]. Type III allorecognition evolved first, followed by Type II and Type I [10], with the amount of physical contact and tissue remodeling before rejection occurs decreasing over evolutionary time. *B. scalaris,* the only known species with Type III allorecognition, occupies the basal position in the botryllid phylogeny [10], followed by two species with Type II allorecognition: *B. primigenus* and *Botryllus schlosseri* [10,54]. Type I allorecognition is found in *Botrylloides* species, which are the most derived in the botryllid phylogeny [10,54]. This evolution from allorecognition mechanisms requiring extensive physical contact between colonies to those requiring limited contact suggests that external allorecognition in botryllids evolved from systems that already existed within individuals: defense against pathogens or stem cell parasitism, self-incompatibility or lineage sorting during development [10].

One botryllid species, *Botryllus horridus,* does not engage in fusion or rejection reactions under natural conditions [50]. While *B. horridus* is an early-branching lineage in the *Botryllus/Botrylloides* phylogeny [54], *B. primigenus* branches earlier and has allorecognition abilities [47]. Therefore, the *B. horridus* lineage must have lost the ability to engage in allorecognition reactions.

Returning to *P.zorritensis,* the second place in the stolidobranch phylogenetic tree where coloniality exists, no one has published on allorecognition in this species. A congener, *Polyandrocarpa misakiensis,* exhibits no allorecognition behaviors [55]. However, *P. misakiensis* and *P. zorritensis* are not closely related; *Polyandrocarpa* is a polyphyletic genus [36]. Because coloniality likely evolved separately in Polyzoinae + Botryllinae and *P. zorritensis,* it would be interesting to learn whether the allorecognition mechanisms in *Symplegma, Botryllus/Botrylloides* and *P. zorritensis* share any similarities at the genetic level, assuming *P. zorritensis* evolved allorecognition.

### 3.2. Order Phlebobranchia

#### 3.2.1. Coloniality in Order Phlebobranchia

The Order Phlebobranchia contains both colonial and solitary species, but each family has a single growth form [23]. The ancestor of the Phlebobranchia was likely solitary, as the majority of the Phlebobranchia are solitary. There are three colonial genera and 58 colonial species within the Phlebobranchia: *Ecteinascidia* (29 species)*, Perophora* (23 species), and *Plurella* (6 species), compared with 36 solitary genera and 278 solitary species [23]. In the genera *Ecteinascidia* and *Perophora* (family Perophoridae)*,* the inner vesicle is derived from the stolonial mesenchymal septum [35,56]. There are no published phylogenetic trees that include *Plurella*, but Kott [57] did not find evidence for a close relationship between *Plurella* and Perophoridae because *Plurella* zooids are embedded in a test rather than being connected by stolons as in Perophoridae. Although the mechanism of budding is unknown in *Plurella,* it is very unlikely to be stolonic as in Perophoridae. Evolutionarily, *Plurella* should be close to the solitary phlebobranch family Ascidiidae [57]. For these reasons, coloniality likely evolved twice in the Phlebobranchia: once in the ancestor of the Perophoridae, and once in the ancestor of the genus *Plurella.*

#### 3.2.2. Allorecognition in Order Phlebobranchia

Within the Order Phlebobranchia, there are two colonial groups: family Perophoridae and Genus *Plurella.* There are no studies of *Plurella* species in an allorecognition context. Within the family Perophoridae, *Ecteinascidia tortugensis, Perophora bermudensis, Perophora japonica, Perophora sagamiensis* and *Perophora viridis* show evidence of allorecognition abilities, whereas *Perophora orientalis* does not undergo natural fusion with either self or non-self-colonies [58,59,60]. Further study of the evolution of allorecognition in the colonial phlebobranchs awaits investigations of allorecognition in a larger number of species, as well as a phylogeny of the family Perophoridae.

### 3.3. Order Aplousobranchia

#### 3.3.1. Coloniality in Order Aplousobranchia

The ancestral aplousobranch was colonial [30]. The species in the Order Aplousobranchia are all colonial, with the interesting exception of the family Diazonidae; genera *Pseudorhopalaea* and *Rhopalaea* are solitary but the genera *Diazona, Pseudodiazona* and *Tylobranchion* are colonial [30,61,62]. Given that the rest of the Aplousobranchia are colonial, the solitary Diazonidae genera must be a reversion back to the ancestral growth form found at the base of the Phlebobranchia/Aplousobranchia clade [30].

**Figure 3:**
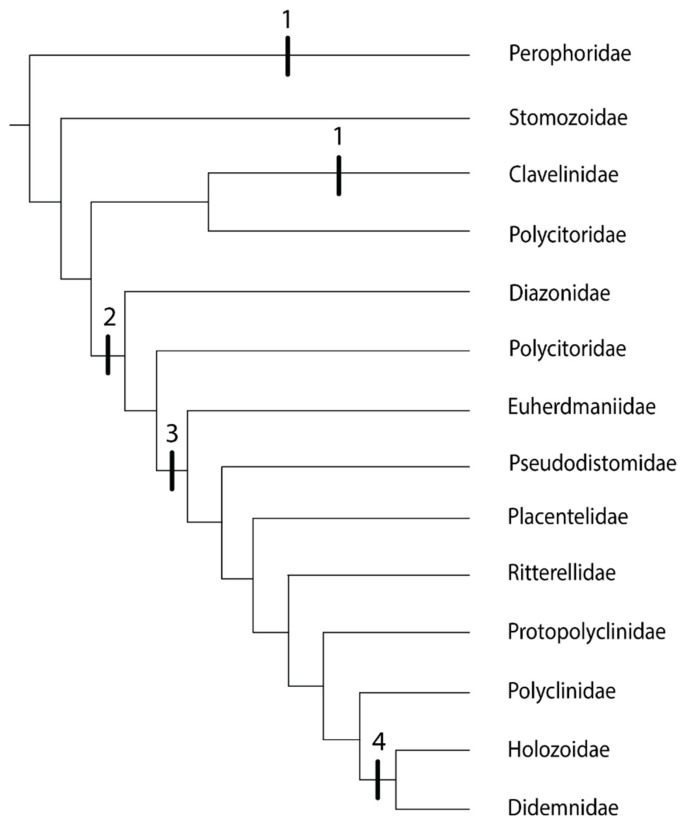
Phylogeny of the Order Aplousobranchia, based on [30,63,65]. The tree includes family Perophoridae (Order Phlebobranchia) as the outgroup. Vertical bars represent budding types. **1**: stolonic; **2**: inner bud vesicles form by epidermal constriction of the abdomen; **3**: inner bud vesicles formed by epidermal constriction of the post-abdomen; **4**: epicardial budding with no constriction of the abdomen or post-abdomen.

In the genera of the family Clavelinidae within the Aplousobranchia for which budding is known (*Clavelina* and *Nephtheis*), the inner vesicles of the buds form from the stolonial mesenchymal septum, the same mechanism as in the phlebobranch family Perophoridae [35]. Both of these families have zooids connected by stolons [63]. However, these two groups are separated by family Stomozoidae (Figure 3). Although budding in Stomozoidae is unknown, there is no evidence that species in this group reproduce by stolonic budding [61]. *Polycitor circes,* a member of the polyphyletic family Polycitoridae, groups with family Clavelinidae and also shows no evidence of stolonic budding [63]. An ancestral aplousobranch with stolonic budding would mean a loss of stolonic budding in Stomozoidae and *P. circes* (Figure 3)*,* so the convergent evolution of this trait in the families Perophoridae and Clavelinidae is the more parsimonious explanation.

Aside from the family Clavelinidae, there are three additional budding modes in the Aplousobranchia, and therefore three more possibilities for the independent evolution of coloniality in this group. The next clades after the family Clavelinidae are family Diazonidae and a clade including the following members of the family Polycitoridae: *Brevicollus, Eudistoma, Exostoma,* and *Polycitor crystallinus* (Figure 3). In these groups, inner bud vesicles form by epidermal constriction of the abdomen when budding type is known [63]. The final clade in the aplousobranch phylogeny experienced two evolutions of coloniality: inner bud vesicles formed by epidermal constriction of the post-abdomen (Figure 3: Euherdmaniidae through Polyclinidae), and then epicardial budding with no constriction of the abdomen or post-abdomen as the most derived budding mechanism (Figure 3: Holozoidae and Didemnidae) [63].

#### 3.3.2. Allorecognition in Order Aplousobranchia

Although studies on this topic are not common in the speciose Order Aplousobranchia, allorecognition occurs across the aplousobranch phylogenetic tree, in the Polycitoridae, Polyclinidae, and Didemnidae. Researchers have not studied the two families that occupy the basal positions in the phylogenetic tree of Aplousobranchia [63], Stomozoidae and Clavelinidae, in a fusion or allorecognition context, so it is hard to speculate whether the ancestral aplousobranch was capable of allorecognition.

In the family Polycitoridae, *Cystodytes dellechiajei* exhibits allorecognition [64]. However, *Cystodytes, Eudistoma* and *Archidistoma,* all polycitorids, fall in different places in the phylogenetic tree [65], so *C. dellechiajei* likely does not represent all members of this polyphyletic family. In the family Polyclinidae, *Aplidium constellatum* [58] and *Aplidium yamazii* [66] exhibit allorecognition. In the family Didemnidae, *Didemnum moseleyi* [47,67] and *Didemnum vexillum* [68,69] exhibit allorecognition. The family Didemnidae is exceptionally speciose, so the behaviors of two species should not be taken to represent the entire family. Four other species in the family Didemnidae: *Didemnum fulgens, Didemnum rodriguesi, Trididemnum solidum,* and *Trididemnum tenerum*, fuse with conspecifics, but colony specificity is an open question [70,71,72,73].

In *Diplosoma listerianum* [74], a lack of colony specificity leads to indiscriminate fusion. Indiscriminate fusion in this species may be explained by loss of alleles in the allorecognition genes if the benefits of fusion are greater than the costs [74]. A potential risk of fusion is that one partner may contribute more to the tunic production than the other partner, but the tunic in *D. listerianum* is thin and insubstantial [74], even compared with other members of the genus.

## 4. Evolution of Allorecognition within the Subphylum Tunicata: Regulating Mating

Researchers have studied the genes/proteins involved in self-incompatibility in three ascidian species: the phlebobranch *C. robusta* [20] and the stolidobranchs *H. aurantium* and *H. roretzi* [21,22]. Some *Ciona intestinalis* and *C. robusta* individuals can self-fertilize in vitro, likely because of the high concentration of sperm used in the experiments [75,76,77]. However, individuals in these species cannot self-fertilize in a laboratory flume and are therefore unlikely to do so in nature [78]. In *C. robusta,* the proposed mechanism of the self-incompatibility system operates by self-recognition [20] and is under genetic control [79,80]. There are two sets of proteins involved: s-Themis A and B (sperm), which act as ligands, and v-Themis A and B (egg), which act as receptors [20]. When s-Themis ligands bind to v-Themis receptors, s-Themis recognizes self v-Themis, s-Themis detaches from v-Themis, and the sperm do not penetrate the vitelline coat [20]. 

In contrast to *Ciona,* species in the stolidobranch genus *Halocynthia* are not self-fertile, even at high sperm concentrations [81]. While researchers have not confirmed genetic control of allorecognition in *Halocynthia*, they have identified candidate molecules [81]. A protein on the surface of the vitelline coat in *H. roretzi*, named HrVC70, binds more effectively to non-self-sperm than to self-sperm [21]. HrVC70 binds to a sperm protein called HrUrabin [82]. While HrUrabin is not highly polymorphic, it seems to be necessary for self-sperm to bind to HrVC70, and also mediates the higher affinity of HrVC70 to non-self-sperm [81]. HaVC80 is a homologous protein to HrVC70 in *H.aurantium* [22].

Experimental fertilizations provide evidence for the existence of self-incompatibility systems in other tunicate species, although the genes and proteins underlying such systems are unknown. In the colonial stolidobranch *B. schlosseri*, classic studies show that sperm from one colony is not released at the same time that eggs in the same colony are mature [83,84], which limits the possibility of self-fertilization. However, if colonies are large enough, systems within the same colony can develop asynchronous development and self-fertilization could occur [78]. Indeed, some colonies in Monterey, California, contained newly ovulated eggs and mature testes releasing sperm at the same time [85]. Through a series of crossing experiments, *B. schlosseri* was shown to limit fertilizations, where the gametes are from the same colony, or from sibling colonies [85]. The fusibility locus was thought to control self-sterility, or the fusibility locus and the self-sterility locus were thought to be tightly linked [85]. Similar results were obtained from *B. primigenus* [86]. However, there was no evidence for allorecognition genotypes influencing sperm–egg interactions in a subsequent study in *B. schlosseri* [4], and the fusibility gene (FuHC) is not expressed in germline tissue in *B. schlosseri* [87]. Researchers have not subsequently investigated self-sterility in *B. schlosseri*, nor have they studied linkages between fusibility and self-sterility in *B. primigenus*.

The phlebobranch *Ciona savignyi* is self-fertile, but self-fertilization takes longer than non-self-fertilization, and non-self-fertilization was 100% in a mixture of self and non-self-sperm [88]. The phlebobranch *Corella willmeriana* is capable of both self-fertilization and outcrossing, and success at self-fertilization varies widely between individuals [89,90]. While this could result from varying levels of self-compatibility, varying levels of inbreeding depression is more likely [89]. The phlebobranchs *Ascidia ceratodes* [85] and *Ascidia mentula* [91] possess a block to self-fertilization as does the stolidobranch *Herdmania momus* [92]. The aplousobranch ascidian *D. listerianum* shows incompatibilities between potential mates [93,94], although these incompatibilities cannot be predicted by genetic relatedness [94].

## 5. Conclusions

In invertebrate animals such as tunicates, allorecognition is linked to coloniality and self-incompatibility mating systems. Within the subphylum Tunicata, coloniality likely evolved independently at least six times: once in the Class Thaliacea, twice in the Phlebobranchia, once in the Aplousobranchia, and twice in the Stolidobranchia. There are two colonial groups within the Phlebobranchia; of these colonial species, five species exhibit allorecognition and one does not. Further study of the evolution of allorecognition in the colonial phlebobranchs awaits investigations of allorecognition in a larger number of species. In the Aplousobranchia, allorecognition seems to be found across the phylogenetic tree, in the families Polycitoridae, Polyclinidae, and Didemnidae. Within the Stolidobranchia, allorecognition evolved either at the base of the Polyzoinae/Botryllinae clade, or separately in each subfamily.

Researchers have studied the genes/proteins involved in self-incompatibility in three ascidian species: the phlebobranch *C.robusta* and the stolidobranchs *H.aurantium* and *H.roretzi*. In *C.robusta*, the proposed mechanism of the self-incompatibility system operates by self-recognition and is under genetic control. There are two sets of proteins involved: s-Themis A and B (sperm), which act as ligands, and v-Themis A and B (egg), which act as receptors. When s-Themis ligands bind to v-Themis receptors, s-Themis recognizes self v-Themis, s-Themis detaches from v-Themis, and the sperm do not penetrate the vitelline coat. In *H. roretzi,* a protein on the surface of the vitelline coat, HrVC70, binds more effectively to non-self-sperm than to self-sperm. HrVC70 binds to a sperm protein called HrUrabin, which seems to be necessary for self-sperm to bind to HrVC70 and also mediates the higher affinity of HrVC70 to non-self-sperm.
